# Two new agaricoid species of the family Clavariaceae (Agaricales, Basidiomycota) from China, representing two newly recorded genera to the country

**DOI:** 10.3897/mycokeys.57.36416

**Published:** 2019-08-21

**Authors:** Ming Zhang, Chao-Qun Wang, Tai-Hui Li

**Affiliations:** 1 State Key Laboratory of Applied Microbiology Southern China, Guangdong Provincial Key Laboratory of Microbial Culture Collection and Application & Guangdong Open Laboratory of Applied Microbiology, Guangdong Institute of Microbiology, Guangdong Academy of Sciences, Guangzhou 510070, China Guangdong Institute of Microbiology, Guangdong Academy of Sciences Guangzhou China

**Keywords:** *
Camarophyllopsis
*, *
Hodophilus
*, phylogenetic analysis, subtropical zone, taxonomy

## Abstract

Two new lamellar species, *Camarophyllopsis olivaceogrisea* and *Hodophilus glaberripes*, of the family Clavariaceae were discovered in the subtropical zone of China. *Camarophyllopsis olivaceogrisea* is morphologically characterized by its hygrophanous basidiomata, greenish gray to dull green pileus, shortly decurrent lamellae, broadly elliptic basidiospores 4–5.5 × 3.5–4.5 μm in size, and cutis-like pileipellis composed of cylindrical cells. *Hodophilus glaberripes* is mainly characterized by its white to brownish pileus, glabrous stipe, slight yam bean smell, broadly elliptic basidiospores 5–6.5 × 4–5 μm in size, and epithelium-like pileipellis composed of inflated cells. Phylogenetic placement of the two species was determined by the combined analyses of a DNA data matrix containing ITS and LSU, and showed that collections of the two species formed two independent lineages in the *Camarophyllopsis* and *Hodophilus* clades respectively. The delimitation of *C. olivaceogrisea* and *H. glaberripes* were evaluated using molecular, morphological, and ecological methods. This is the first report of the genera *Camarophyllopsis* and *Hodophilus* in China.

## Introduction

Clavariaceae Chevall. (Agaricales, Basidiomycota) is a genetically monophyletic but morphologically diverse family. Members of this family show variations in the macromorphology of their sporocarps and are pendant-hydnoid, cylindrical, clavate, coralloid, resupinate, pileate, or lamellate-stipitate (Larsson et al. 2004; Dentinger and McLaughlin 2006; Matheny Curtis et al. 2006; Larsson 2007; Birkebak et al. 2013). Generally, Clavariaceae was known as a coral fungal group, including club-like (clavarioid) genera such as *Clavaria* Vaill. ex L., and *Clavulinopsis* Overeem, and branch-form (coralloid) genera, such as *Ramariopsis* (Donk) Corner (Birkebak et al. 2013, 2016). However, some agaricoid genera, such as *Camarophyllopsis* Herink, *Hodophilus* R. Heim ex R. Heim and *Lamelloclavaria* Birkebak & Adamčík, showed phylogenetic affinity within Clavariaceae (Matheny Curtis et al. 2006; Birkebak et al. 2013), thus have been added to the family based on phylogenetic analyses in recent years (Birkebak et al. 2016).

*Camarophyllopsis* can be easily distinguished from other genera in the family by its small agaricoid basidiomata, hygrophanous pileus, subglobose to broadly ellipsoidal basidiospores, and epithelium pileipellis composed of chains of erect, ascending or repent, subcylindrical to ellipsoid terminal cells (Arnolds 1986; Young 2005). *Hodophilus* differs from *Camarophyllopsis* in the hymeniderm pileipellis composed of typically perpendicular, broadly inflated, globose, and obpyriform to sphaero-pendunculate terminal elements (Birkebak et al. 2016). *Lamelloclavaria* can be distinguished from the above genera by its rimulose non-hygrophanous pileus and cutis pileipellis (Birkebak et al. 2016).

In this study, some agaricoid collections were identified in China. Morphological observation and phylogenetic analyses confirmed that they are two novel taxa in the genera *Camarophyllopsis* and *Hodophilus*. This is the first report of these two genera in China.

## Materials and methods

### Morphological studies

Photographs of basidiomata were taken in type localities when collected. Macro-morphological characteristics were recorded for fresh specimen. Specimens were dried and then deposited in the Guangdong Institute of Microbiology (GDGM). Methods used for morphological descriptions were followed Zhang et al. (2015). Colors were recorded and described in general terms according to the method of Kornerup and Wanscher (1978). Microstructures were observed from rehydrated materials, and the notation “basidiospores (n/m/p)” indicates that the measurements were conducted for n basidiospores from m basidiomata of p collections. Line drawings were prepared by free hand.

### DNA extraction, PCR amplification, and sequencing

Total genomic DNA of each voucher specimen was extracted from silica-gel-dried materials using the Sangon Fungus Genomic DNA Extraction kit (Sangon Biotech, Shanghai, China) according to the manufacturer’s instructions. Primer pairs ITS1/ITS4 (White et al. 1990) and LR0R/LR5 (Vilgalys and Hester 1990) were used to amplify the internal transcribed spacer (ITS) region and the large subunit nuclear ribosomal RNA (nrLSU) region, respectively. PCR protocol and sequencing were conducted following the method of Zhang et al. (2015).

### Phylogenetic analyses

Newly generated sequences, related sequences used in previous studies (Adamčík et al. 2018) and a few sequences retrieved from GenBank by a Blast search were used to reconstruct phylogenetic trees. Detailed information on the newly sequenced samples, including the taxon names, voucher numbers, localities and GenBank accession numbers, is shown in Table 1.

**Table 1.** Information on newly generated DNA sequences used in this study.

Taxon	Voucher	Country	ITS	LSU
*Camarophyllopsis olivaceogrisea*	GDGM44497	China	MK894563	MK894551
	GDGM44519	China	MK894564	MK894552
*Camarophyllopsis* sp.	GDGM44501	China	MK894565	MK894553
*Hodophilus glaberripes*	GDGM45940	China	MK894566	MK894554
	GDGM52374	China	MK894567	MK894555
	GDGM52530	China	MK894568	–
	GDGM52545	China	MK894569	MK894556
	GDGM52583	China	MK894570	MK894557
	GDGM55689	China	MK894571	MK894558
	GDGM70329	China	MK894572	MK894559
	GDGM70331	China	MK894573	MK894560
	GDGM72434	China	MK894574	MK894561
	GDGM72518	China	MK894575	MK894562

ITS and LSU sequences were respectively aligned using Clustal X v1.81 (Thompson et al. 1997) and manually modified where necessary in Bioedit v7.0.9 (Hall 1999), and a combined matrix of ITS and LSU sequences was obtained. The combined dataset was then analyzed using RAxML v7.2.6 (Stamatakis 2006) and MrBayes v3.1.2 software (Ronquist and Huelsenbeck 2003) for maximum likelihood (ML) and Bayesian inference (BI) analyses, respectively. For both BI and ML analyses, the substitution model for the two gene partitions was individually determined using the Akaike Information Criterion (AIC) complemented in MrModeltest v2.3 (Nylander 2004). For ML analysis, all parameters were kept at default values, except for choosing the GTRGAMMAI model, and statistical support was obtained using rapid nonparametric bootstrapping with 1000 replicates. BI analysis using selected models and 4 chains was conducted by setting the number of generations to 3 million and stoprul command with the stopval value set to 0.01. Trees were sampled every 100 generations. The first 25% of generations were discarded as burn-ins and posterior probabilities (PP) were calculated from the posterior distribution of the retained Bayesian trees.

## Results

### Molecular phylogenetic results

**Figure 1.**
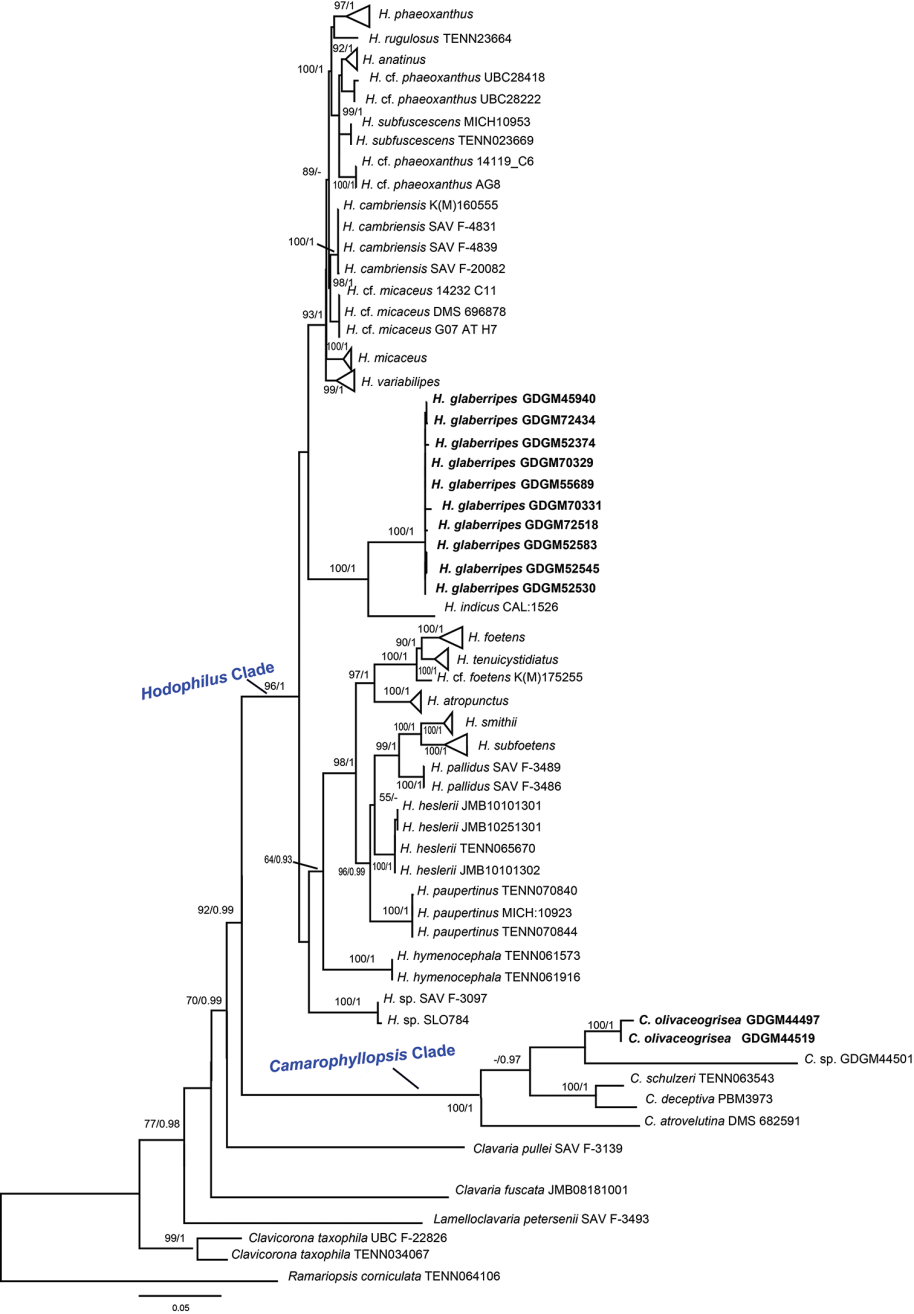
Phylogenetic placements of *Camarophyllopsis olivaceogrisea* and *Hodophilus glaberripes* inferred from the combined ITS and LSU dataset using RAxML. *Ramariopsis corniculata* was selected as an outgroup. The lineages with new species were shown in bold. BS ≥ 50% and PP ≥ 0.90 were indicated around the branches.

For phylogenetic analyses, 25 sequences (13 ITS and 12 LSU) were newly generated from 13 collections, 219 related sequences (123 ITS and 96 LSU) were retrieved from GenBank, and *Ramariopsis corniculata* (Schaeff.) R.H. Petersen selected as an outgroup based on previous studies (Birkebak et al. 2016; Adamčík et al. 2016, 2018). The combined matrix of 137 samples with 1614 nucleotide sites was constructed for phylogenetic analyses and the final alignment was submitted to TreeBASE (Submission ID 24440). SYM+I and SYM+G were chosen as the best substitution models for ITS and LSU, respectively. The ML and BI analyses generated nearly identical tree topologies with minimal variation in statistical support values; thus, a ML tree was selected for display (Figure 1).

The tree topologies generated in this study are similar to those obtained by Adamčík et al. (2018) and are therefore not described in detail here, except for the results relevant to the two new species. The two monophyletic genera *Camarophyllopsis* and *Hodophilus* were highly supported (Figure 1). Two collections (GDGM44497 and GDGM44519) formed an independent lineage with strong statistical support (BS = 100, PP = 1), located within the *Camarophyllopsis* clade, and presented as a sister group to a collection numbered as GDGM44501 from China (with low statistical support). Ten collections (GDGM45940, GDGM52374, GDGM52530, GDGM52545, GDGM52583, GDGM55689, GDGM70329, GDGM70331, GDGM72434 and GDGM72518) were grouped together with strong statistical supports (BS = 100, PP = 1) and formed an independent lineage in the *Hodophilus* clade, and were revealed as a sister group to *H. indicus* K.N.A. Raj, K.P.D. Latha & Manim. with significant statistical support (BS = 100, PP = 1).

## Taxonomy

### Camarophyllopsis olivaceogrisea

Taxon classificationFungiAgaricalesClavariaceae

Ming Zhang, C.Q. Wang & T.H. Li sp. nov.

5A0FF714F3665916AD0EB0738BAE6B88

MB831122

Figs 2a–b , 3

#### Etymology.

The epithet “*olivaceogrisea*” refers to the olive-gray pileus color.

#### Diagnosis.

This new species is morphologically distinguished from other taxa in the genus by its smaller basidiomata, greenish gray to dull green pileus, white and short decurrent lamellae, and broadly elliptic basidiospores.

#### Type.

CHINA. Guizhou Province: Leishan County, Leigongshan National Nature Reserve, alt. 1260 m, 22 July 2014, M. Zhang (holotype: GDGM44519!).

#### Description.

Basidiomata small-sized. Pileus 7–12 mm broad, hemispherical, convex to plano-convex at first, then gradually applanate, becoming depressed at disc when mature, non-striate to weakly striate; margin slightly inflexed at first, soon straight, slightly crenate or lacerate when mature; surface matt, velvety, hygrophanous, greenish gray (or olive gray) to dull green (27D 2–30D2, 27D3–30D3, 27E2–30E2, 27E3–30E3), often paler at margin. Flesh 1–3 mm thick in the stipe, white to grayish white, unchanging when exposed. Lamellae 1–2 mm deep, L = 20–34, l = 1–3, short to moderately decurrent, white to weakly grayish white (1A1–1B1) at first, white to weakly greenish white (27A2–30A2) when mature, unchanging when bruised; edge entire, concolorous with the sides. Stipe 13–25 × 1.5–2.5 mm, central, cylindrical and becoming narrower downwards; glabrous and shiny, hardly tomentulose or pruinose, hygrophanous, white to greenish white at first (28A1–28A2, 29A1–29A2), becoming greenish white to light greenish gray (28A2–28B2, 29A2–29B2) when mature and in dry condition. Odor none. Taste mild.

Basidiospores [60/2/2] 4–5.5(–6) × 3.5–4.5(–5) μm, av. 4.6 × 3.8 μm, Q = (1.12)1.14–1.28(1.43), av. Q = 1.21 ± 0.08, broadly ellipsoid, hyaline, smooth, inamyloid, thin-walled. Basidia 4-spored, occasionally 2-spored, (10–)15–26(–30) × 5–7 μm, av. 24.5 × 5.8 μm, hyaline, narrowly clavate, attenuated and ﬂexuous toward base, sterigmata up to 4 μm long. Basidioles cylindrical to narrowly clavate, often ﬂexuous, obtuse, (18–)20–37.5(–40) × 6–8 μm, av. 23 × 6.8 μm. Pleurocystidia absent. Marginal cells on the lamellar edges not well differentiated, similar to basidioles on lamellar sides. Lamellar trama composed of sub-parallel or occasionally interwoven and irregularly inflated hyphae (23–)35–50(–104) × 4–8(–10) μm, av. 46.5 × 7 μm. Pileipellis a cutis of numerous repent branched hyphal 4–8 μm wide, with terminal chains of ellipsoid or cylindrical cells. Pileus trama composed of cylindrical and occasionally branched hyphae (23–)35–50(–70) × (4–)6–10 μm, av. 45.5 × 7.6 μm. Stipitipellis formed of parallel, thin-walled and narrow hyphae 3–8 μm diam. Caulocystidia not observed. Clamp connections absent in all tissues.

**Figure 2.**
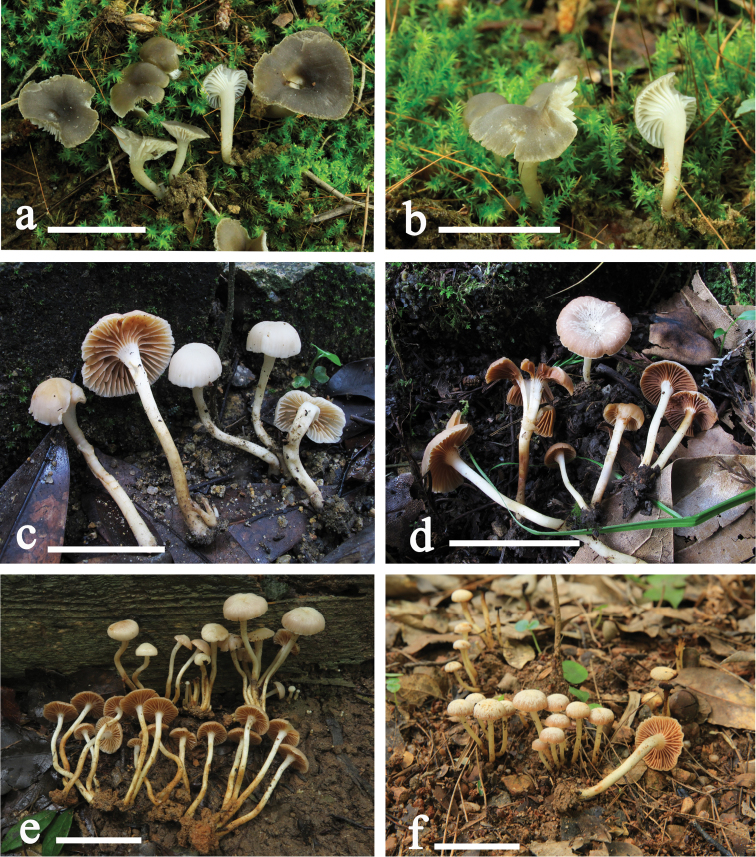
**a, b** Basidiomata of *Camarophyllopsis olivaceogrisea* (GDGM44519, holotype) **c–f** basidiomata of *Hodophilus glaberripes* (e. GDGM72518, holotype). Scale bars: 20 mm (**a, b**); 50 mm (**c–f**).

**Figure 3.**
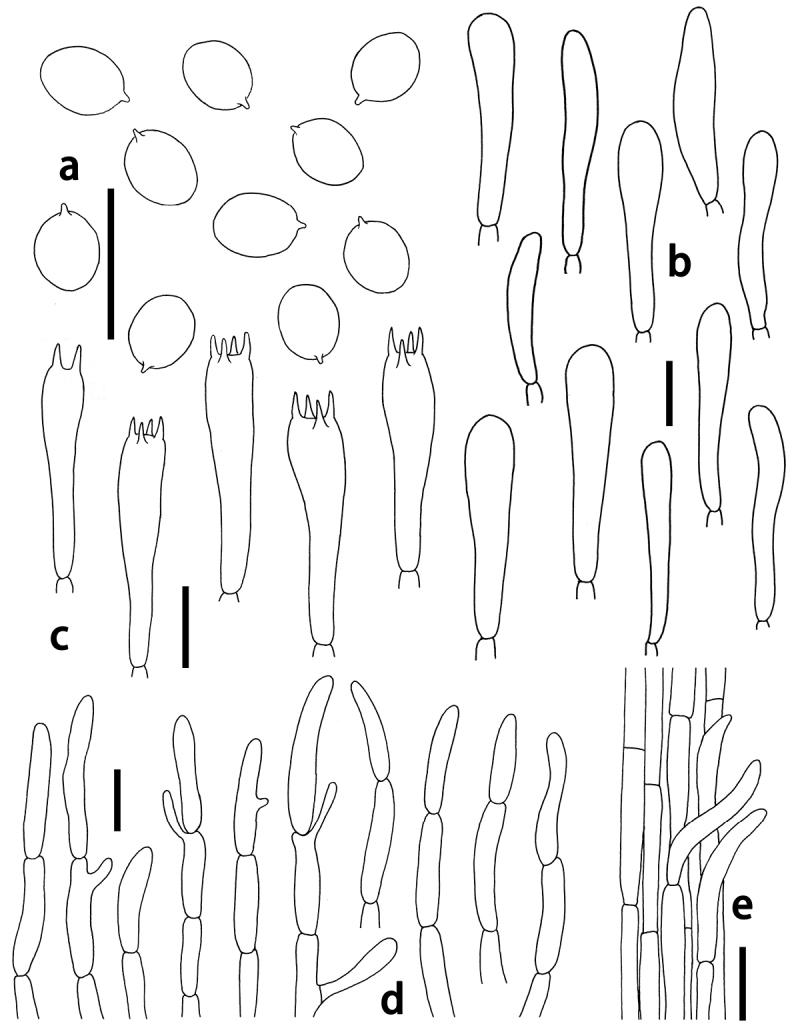
*Camarophyllopsis olivaceogrisea*. **a** Basidiospores **b** basidioles **c** Basidia **d** hyphal terminations in pileipellis **e** stipitipellis. Scale bars: 10 μm (**a–c**); 20 μm (**d, e**).

**Figure 4.**
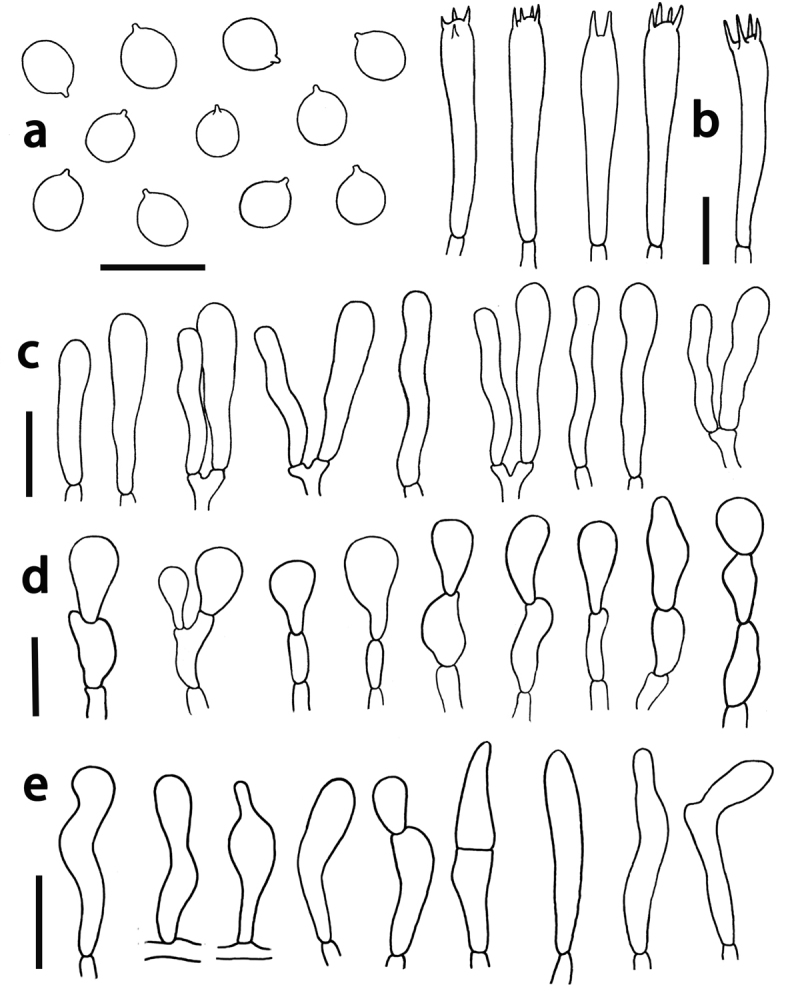
*Hodophilus glaberripes*. **a** Basidiospores **b** basidia **c** basidioles **d** hyphal terminations in pileipellis e hyphal terminations in stipitipellis. Scale bars: 10 μm (**a–c**); 20 μm (**d, e**).

#### Habit, ecology and distribution.

Solitary, scattered on soil in mixed forests; currently only known from the Guizhou Province of China.

#### Additional specimens examined.

CHINA. Guizhou Province: Leishan County, Leigongshan National Nature Reserve, alt. 1120 m, 22 July 2014, J. Xu (GDGM44497).

### Hodophilus glaberripes

Taxon classificationFungiAgaricalesClavariaceae

Ming Zhang, C.Q. Wang & T.H. Li sp. nov.

4D2E8D86CC955AD39A7B278A7B1A7CA1

MB831125

Figs 2c–f , 4

#### Etymology.

The epithet refers to the glabrous stipe.

#### Diagnosis.

This species is easily distinguished from other species in the genus *Hodophilus* by its larger basidiomata, white, brownish orange to brown pileus, glabrous stipe, slightly yam bean smell and broadly elliptic basidiospores.

#### Type.

CHINA. Guangdong Province: Shaoguan City, Danxiashan National Nature Reserve, alt. 240 m, 10 May 2018, M. Zhang (holotype: GDGM72518!).

#### Description.

Basidiomata small to medium-sized. Pileus 15–50 mm broad, hemispherical, convex to plano-convex at first, then becoming broadly convex or plano-convex but hardly fully expanded to plane, often depressed at disc when old; white to yellowish white at first, then gradually becoming orange white, pale yellow, pale orange, brownish orange, light brown, brown to reddish brown (5A2, 3A3–5A3, 5C4–7C4, 5D5–9D5) when mature and dry, hygrophanous; margin first slightly inflexed, soon straight, slightly crenate when mature, non-striate or indistinctly translucently striate up to one third when wet; surface matt, velvety and later with fine and darker granules or pruina, at first even, but becoming rugose or rough towards the center when mature, often concentrically cracked in dry conditions. Flesh 0.5–2 mm thick in half radius of the pileus, white, pinkish white to pale beige; Lamellae 3–5 mm deep, distant to subdistant, L = 21–32, l = 1–3, short decurrent, notched, orange white to pinkish white (5A2–10A2) when young, brownish orange, light brown, reddish brown to brownish red (5C4–7C4, 5D6–10D6) when mature, unchanging when bruised; edge entire, concolorous or slightly paler than lamella sides. Stipe (50) 80–100 × 3–5 mm, cental, usually flexuous, cylindrical and slightly narrower downwards; glabrous smooth and shiny, hygrophanous, white to yellowish white at first, becoming pale yellow to pale orange when mature and in dry condition. Odor none or slight yam bean smell, taste mild.

Basidiospores [210/9/9] (4.5)5–6.5(7) × 4–5(5.5) μm, av. 5.9 × 4.7 μm, Q = (1.0)1.11–1.37(1.4), av. Q = 1.20 ± 0.11, broadly ellipsoid to subglobose, hyaline, smooth, inamyloid, thin-walled. Basidia 4-spored, occasionally 2-spored, (32–) 36–46(–66) × (4–)4.5–6(–7) μm, av. 39.5 × 5.9 μm, tenuated and flexuous towards base, with sterigmata up to 7 μm long. Basidioles cylindrical to narrowly clavate, obtuse, often flexuous, (31–)34–42(–60) × (4–)6–8(–10) μm, av. 40.5 × 6.9 μm. Pleurocystidia absent. Marginal cells on the lamellar edges usually not well differentiated, similar to basidioles on lamellar sides. Lamellar trama composed of sub-parallel or occasionally interwoven and irregularly inflated branched hyphae with elongate cells (38–)52–98(–160) × (4–)6–14(–20) μm, av. 88 × 9.5 μm. Subhymenium poorly developed. Pileipellis a transition from hymeniderm to epithelium, with hyphal elements 3–10 μm wide, thin-walled, hyaline, terminations usually composed of 1–3 inflated cells; terminal cells obpyriform, subglobous or ellipsoid, rarely sphaero-pedunculate or broadly clavate, (15–)19–46(–50)× (7–)12–22(–30) μm, av. 38.5 × 18 μm. Pileus trama composed of subparallel hyphae (34–)46–89(–130) × (4–)5.5–10 μm, av. 74 × 7.6 μm. Stipitipellis formed of parallel, thin-walled and narrow hyphae 3–6 μm diam. Caulocystidia usually in dense fascicles or patches, thin-walled, repent or ascending; with terminal cells mainly clavate, occasionally subcapitate or obpyriform, obtuse, often pedicellate and flexuous, measuring (18–)22–53(–60)× (4–)5.5–13 μm, av. 43 × 7.5 μm. Clamp connections absent in all tissues.

#### Habit, ecology and distribution.

Solitary, scattered on soil in broadleaf forests and mixed forests; currently only known from China.

#### Additional specimens examined.

CHINA. Guangdong Province: Huizhou City, Xiangtoushan National Nature Reserve, alt. 640 m, 18 May 2016, H. Huang (GDGM45940); Shaoguan City, Nanling National Nature Reserve, 800 m, 29 July 2017, M. Zhang (GDGM70329 and GDGM70331); Shaoguan City, Danxiashan National Nature Reserve, 200 m, 27 April 2019, X.R. Zhong (GDGM76367 and GDGM76337), J.P. Li (GDGM76300); Jiangxi Province: Jinggangshan Botanical Garden, 884 m, 20 June 2016, H. Huang (GDGM52374), Z.P. Song (GDGM52545); same location, 21 June 2016, Z.P. Song (GDGM52530 and GDGM52583); Hunan Province: Chenzhou City, Jiulongjiang National Forest Park, alt. 230 m, 14 May 2018 X. R. Zhong (GDGM55689).

## Discussion

### Key to the species of *Camarophyllopsis* and *Hodophilus*

**Table:** 

1	Basidiomata agaricoid; pileipellis an epithelium composed of chains of subcylindrical to ellipsoid terminal elements	**2 *Camarophyllopsis***
–	Basidiomata agaricoid; pileipellis a hymeniderm composed of broadly inflated, globose, and obpyriform to sphaero-pendunculate terminal elements	**12 *Hodophilus***
2	Pileus diameter usually < 30 mm	**3**
–	Pileus diameter usually ≥ 30 mm	**11**
3	Pileus hygrophanous or subhygrophanous	**4**
–	Pileus not hygrophanous	**5**
4	Pileus greenish gray to dull green; lamellae white, decurrent; basidiospores 4–5.5 × 3.5–4.5 μm	***C. olivaceogrisea***
–	Pileus rugulose, buffy brown to dark hazel; lamellae decurrent, pinkish to hazel; stipe olive brown or grayer; basidiospores globose av. 4 × 5 μm	***C. rugulosoides***
5	Lamellae always with pink tinct, pale pink to pink, decurrent; pileus pale pink to whitish, matt; stipe pale pink to whitish; basidiospores av. 7 × 4.5 μm	***C. roseola***
–	Lamellae without pink tinct	**6**
6	Lamellae white, unchanging when mature	**7**
–	Lamellae whitish to grayish or brownish	**9**
7	Basidiospores < 6 μm; pileus brownish to dark brown, subtomentosus, depress in central when mature; stipe concolorous with pileus or slightly faded	***C. atrovelutina***
–	Basidiospores usually ≥ 6 μm	**8**
8	Pileus gray, manifestly cleaving at margin when dry; stipe white; basidiospores 5.5–8.5 × 4–5.5 μm	***C. leucopus***
–	Pileus gray, sub-velvety; stipe pale gray; basidiospores 7–8 × 5.5–6.5 μm	***C. tetraspora***
9	Basidiospores ≥ 7 μm; pileus fuliginous brown, subfibrillose to fibrillose, infundibuliform; lamellae deep decurrent; stipe brown	***C. araguensis***
–	Basidospores < 7 μm	**10**
10	Pileus dark brown, subtomentosus; lamellae light grayish, decurrent; stipe white, smooth, basidiospores 5–6.5 × 4–5 μm	***C. albipes***
–	Pileus yellowish cinnamon to brownish cinnamon or chocolate gray, silky-tomentose or velvet; lamellae decurrent, whitish to grayish or brownish; stipe concolorous with pileus; basidiospores 4–5 × 4–4.5 μm	***C. schulzeri***
11	Pileus brownish gray to grayish brown; lamellae adnate to subdecurrent, white; stipe smooth, white; basidiospores 5–7 × 3.5–4.5 μm	***C. pedicellata***
–	Pileus pearl gray to drab, often rivulose-cracking; lamellae light gray; stipe light gray, slightly longitudinally fibrillose-striped; basidiospores 5–7 × 3.5–5.2 μm	***C. dennisiana***
12	Basidiomata with a napthalene or an unpleasant odor	**see Adamčík et al. (2016, 2017)**
–	Basidiomata without napthalene odor	**13**
13	Stipe with dark dots on surface	**14**
–	Stipe never with dark dots on surface	**16**
14	Lamellae white to grayish white, pileus pale brown; basidiospores 4.5–6 × 4.0–5 μm, pileipellis an epithelium composed of globose to pyriform elements	’***C. kearneyi***’
–	Lamellae with orange, brown or light brown tinct	**15**
15	Pileus yellowish gray to brown when fresh, light brown, orange gray to beige when mature; lamellae beige or orange gray at first, changing to brownish orange to yellowish brown when mature, basidiospores 4.6–5.4 × 3.5–4.2 μm	***H. atropunctus***
–	Pileus dark brown to pale brown; lamellae light brown to grayish brown when young, changing brown to dark brown when mature; basidiospores 4.8–5.4 × 3.9–4.5 μm	***H. variabilipes***
16	Stipe with yellow tinct	**see Adamčík et al. (2018)**
–	Stipe without yellow tinct	**17**
17	Species has south hemisphere distribution, pileus creamy buff-brown or pinkish fawn; lamellae decurrent, pale pinkish; basidiosproes 4.5–6.0 × 4.5–5.5 μm; pileipellis an epithelium of globose or pyriform elements; known from Australia	’***C. darminensis***’
–	Species has north hemisphere distribution	**18**
18	Pileus yellowish white, brownish orange to reddish brown; lamellae orange white to brownish red; basidiospores 5–6.5 × 4–5 μm; known from China	***H. glaberripes***
–	Pileus grayish brown to brownish orange; lamellae subdecurrent, pale orange; stipe grayish orange, glabrous; basidiospores 4–5 × 3–5 µm; known from India	***H. indicus***

Phylogenetic relationships of the genera within Clavariaceae have been investigated in several studies, and *Camarophyllopsis* and *Hodophilus* were well supported as two independent groups at generic level (Birkebak et al. 2013, 2016; Adamčík et al. 2017, 2018). In the present study, phylogenetic analyses based on ITS and LSU showed that the two clades *Camarophyllopsis* and *Hodophilus* were well supported with high phylogenetic values (BS/BPP = 100/1), and collections from China formed two strongly supported terminal branches in the two clades. The sequences generated in this study did not match any previously described sequences, validating with strong support the recognition of *C. olivaceogrisea* and *H. glaberripes* as two distinct species based on their phenotypic features.

According to the phylogram (Figure 1), *C. olivaceogrisea* nested well into the *Camarophyllopsis* clade and formed a sister group of an unidentified Chinese collection (GDGM44501) with low statistical support. Because of the low number of specimens, GDGM44501 was not described here, but it can be easily separated from *C. olivaceogrisea* by branch distance. The other three species in the phylogenetic tree, *C. atrovelutina*, *C. deceptive* and *C. schulzeri*, also can be easily separated from *C. olivaceogrisea*. The closest relatives of the new species remain unresolved in this study because of the lack of significant statistical supports and the few available sequences of *Camarophyllopsis* used in phylogenetic analysis.

In the *Hodophilus* clade, *H. glaberripes* is closely related to *H. indicus* K.N.A. Raj, K.P.D. Latha & Manim., and together formed a well-supported branch, which is a sister clade to the yellow stipe clade (or *H. micaceus* superclade) as defined by Adamčík et al. (2017, 2018), but with limited statistical support. However, the recently described Indian species, *H. indicus*, show smaller and brownish orange basidiomata, subdecurrent and pale orange lamellae, and slightly smaller basidiospores (4–5 × 3–5 μm) (Crous et al. 2017).

Morphologically, the most distinctive features of *C. olivaceogrisea* are the small basidiomata with a greenish gray to dull green pileus, white and decurrent lamellae, broadly ellipsoid basidiospores, narrowly clavate basidia, and a cutis pileipellis composed of chains of cylindrical cells. *Camarophyllopsis microspora* (A.H. Sm. & Hesler) Bon is similar to *C. olivaceogrisea* to some extent. However, *C. microspora*, originally reported in Michigan, differs on account of its fuscous pileus and stipe, dark grayish context and smaller basidiospores (4–4.5 × 2.5–3 μm) (Hesler and Smith 1963). Considering species with a pileus diameter of 10–30 mm, *C. olivaceogrisea* is similar to *C. albipes* and *C. leucopus* (Singer) Boertm. However, *C. albipes* mainly differs on account of its brown and subtomentose pileus, grayish lamellae with slight veins at the margin, robust stipe, and slightly larger basidiospores (5–6.5 × 4–5 μm) (Singer 1973); *C. leucopus* mainly differs on account of its gray and sulcate pileus and larger basidiospores (5–8.5 × 4–5.5 μm) (Singer 1973).

*Hodophilus glaberripes* is characterized by its larger basidiomata, hygrophanous pileus with white, brownish orange to brown color, glabrous stipe, larger and broadly elliptic basidiospores, epithelium-like pileipellis with obpyriform or subglobous terminal cells, and slightly yam bean smell. The combination of these characteristics makes *H. glaberripes* easily distinguishable from other members of the genus. *Hodophilus glaberripes* is somewhat similar to *H. albofloccipes* (Kovalenko, E.F. Malysheva & O.V. Morozova) Looney and Adamčík, *H. anatinus* Dima, Adamčík & Jančovičová, *H. subfoetens* Adamčík, Jančovičová & Looney, and *H. pallidus* Adamčík, Jančovičová & Looney in morphology. However, *H. albofloccipes* mainly differs by its smaller baisdiomata, ochre or ochre yellow to pale olives pileus, yellow to brownish stipe covered with white pruina or squamula, smaller basidiospores (4–5.7 × 3.5–5 μm), and naphthalene-like odor (Kovalenko et al. 2012); *H. anatinus* differs by its smaller basidiomata, grayish brown pileus, grayish yellow to brown stipe, and smaller basidiospores (4.8–5.5 × 3.8–4.4 μm) (Adamčík et al. 2018); *H. subfoetens* differs by its smaller and grayish brown to brownish black basidiomata, with a naphthalene odor, and smaller basidiospores (5–5.7 × 3.9–4.5 μm) (Adamčík et al. 2017a); *H. pallidus* differs by its smaller basidiomata with a strong naphthalene odor, orange-gray to grayish orange pileus, orange gray to orange brown stipe, and smaller basidiospores (5.1–5.7 × 3.9–4.6 μm) (Adamčík et al. 2017a).

Ecologically, very little is known about the ecology of *Camarophyllopsis* species, as for most species only a few verified collections are known and little molecular data is available. *Camarophyllopsis* species are widely distributed in the southern and northern hemispheres, from tropical zones to cool temperate zones, and in monsoon forest, bushy forest, and grassland habitats, and some species have been shown to be saprotrophic (Young 1999, 2005; Boertmann 2002; Kovalenko et al. 2012). *Camarophyllopsis olivaceogrisea* was collected from subtropical regions in southwest China at altitudes of over 1000 m, and is typically found in wet areas with moss under mixed forest, which is mainly dominated by broadleaf plants (*Castanopsis* spp., *Fagus* spp. and *Schima* spp.) with few conifer (*Pinus massoniana* Lamb. and *Pinus* spp.). This study expands the geographic distribution of the genus *Camarophyllopsis* to China.

*Hodophilus* taxa were mainly reported in temperate to boreal zones of the northern hemisphere and can be found in forest, bushy forest margin, grassland and bare soil habitats (Adamčík et al. 2016, 2017a, 2017b, 2018), and a recent study reported a new tropical distribution in India (Crous et al. 2017). In this study, collections of *H. glaberripes* were distributed from 23°N to 26°N in the subtropical zone of southern China and at altitudes of 200–800 m, mostly occurred in the margin of broadleaf forest (mainly dominated by Fagaceae, Hamamelidaceae and Theaceae plants) and mixed forest (dominated by broadleaf tree mixed with few conifer as *Pinus massoniana* Lamb., *Pinus* spp. and *Cunninghamia* spp.), commonly along the sides of cement road in the forest and preferentially in heavy clay soil to humus. This study revealed an expanded geographic distribution of *Hodophilus* species to subtropical regions.

## Supplementary Material

XML Treatment for Camarophyllopsis olivaceogrisea

XML Treatment for Hodophilus glaberripes
